# Insomnia Symptoms Moderate the Relationship Between Perseverative Cognition and Backward Inhibition in the Task-Switching Paradigm

**DOI:** 10.3389/fpsyg.2020.01837

**Published:** 2020-08-05

**Authors:** Andrea Ballesio, Silvia Cerolini, Mariacarolina Vacca, Fabio Lucidi, Caterina Lombardo

**Affiliations:** ^1^Laboratory of Clinical Psychology and Psychophysiology, Department of Psychology, Sapienza University of Rome, Rome, Italy; ^2^Department of Social and Developmental Psychology, Sapienza University of Rome, Rome, Italy

**Keywords:** perseverative cognition, insomnia, task switching, inhibition, flexibility

## Abstract

Perseverative cognition (PC), that is, the continuous cognitive representation of uncontrollable threats, is known to dampen executive control processes in experimental paradigms. Similarly, PC has been shown to impair sleep and to be implicated in the exacerbation of insomnia, which may in turn contribute to the disruption of executive functions. The interactions between PC and insomnia in influencing executive functions, however, have never been tested to date. In the present study, we explored whether insomnia symptoms may moderate the associations between PC and disrupted executive functions, with the hypothesis to find a stronger relationship between these variables at increasing levels of insomnia. Fifty participants completed measures of trait PC and insomnia severity in the previous month and also completed a computerized task-switching paradigm assessing backward inhibition, switch cost, and accuracy. Prior to the task switching, participants completed a measure of state rumination in order to control for the effects of state PC on cognitive performance. Results show that trait PC was significantly correlated with higher insomnia symptoms and state rumination and marginally correlated with lower backward inhibition and longer switch cost. Moreover, insomnia severity moderated the relationship between trait PC and backward inhibition after controlling for the effects of state rumination; that is, the relationship between PC and inhibitory deficits was stronger in those with higher versus lower levels of insomnia symptoms. Findings suggest the need to better elucidate the associations between PC, insomnia, and executive functioning in clinical samples and longitudinal designs.

## Introduction

Perseverative cognition (PC), also referred to as repetitive negative thinking (e.g., Ehring et al., 2011; Law and Tucker, 2018) or perseverative negative thinking (e.g., Trick et al., 2016), is defined as the repeated or chronic activation of the cognitive representation of one or more psychological stressors. Worry and rumination are often recognized as the two main manifestations of PC. Worry is traditionally defined as a chain of negatively affect-laden thoughts and images related to relatively uncontrollable future outcomes, and it is traditionally associated with generalized anxiety (Borkovec et al., 1983). Rumination is conceptualized as repetitive thoughts about the origins and consequences of one’s personal concerns or negative mood, and it is traditionally studied in the context of depression (Nolen-Hoeksema et al., 2008). Although different in their content, rumination and worry share common underlying cognitive and emotional processes, including intrusiveness, repetitiveness and uncontrollability of the thought, and negative affectivity (Ehring et al., 2011). Moreover, PC has been associated with negative health outcomes. For instance, meta-analytic evidence shows that PC is associated with alterations in cardiovascular, autonomic, and endocrine systems activity (Ottaviani et al., 2016). Additionally, PC tendency is considered a risk factor for developing mental disorders, including depression (Raes, 2012), anxiety (Ruscio et al., 2011), drinking behavior (Caselli et al., 2010), psychosis (Hartley et al., 2017), and to be involved in suicide ideation and behavior (Law and Tucker, 2018). Furthermore, because the experience of PC involves the difficulty to inhibit negative stimuli (e.g., a negative thought) or to shift attention away from them, PC has been associated with deficits in executive functions, and especially with the performance in cognitive tasks requiring attention switching and inhibitory abilities (see Yang et al. (2016) for a meta-analysis). For instance, high trait perseverators are shown to perform poorer on switching tasks compared to nonperseverators (e.g., Beckwé et al., 2014). Moreover, experimental and longitudinal findings confirmed that the induction of PC is associated with performance lapses in tasks requiring switching and inhibitory abilities (Whitmer and Gotlib, 2012; Connolly et al., 2014). Also, PC has been associated with disturbed sleep and insomnia symptoms (i.e., difficulties falling asleep and/or maintaining sleep) in ecological momentary assessments (Lancee et al., 2017) and laboratory (Zoccola et al., 2009; Ballesio et al., 2020) studies. Interestingly, insomnia has been associated with poor executive functions. Insomnia is the most common sleep disorder, with epidemiological studies estimating the prevalence of the insomnia diagnosis up to 20% in Europe (Chaput et al., 2018). Insomnia is defined by difficulties falling asleep and/or maintaining sleep accompanied by significant impairment in daytime functioning, such as fatigue, emotion dysregulation, and cognitive impairment (American Psychiatric Association, 2013). Importantly, insomnia is highly prevalent among those with mental disorders (American Psychiatric Association, 2013), and recent meta-analysis highlighted that insomnia is a risk factor for the development of depression, anxiety, alcohol use, and psychotic disorders (Hertenstein et al., 2019). With respect to cognitive impairment of insomnia, a recent meta-analysis comparing the cognitive performance of individuals with insomnia and good sleepers showed that insomnia is associated with moderate inhibitory (*d* = −0.32) and switching (*d* = −0.30) deficits (Ballesio et al., 2019). Therefore, it is reasonable to hypothesize that PC may interact with insomnia in influencing inhibitory and switching capacities; that is, the effects of PC on inhibitory and switching capacities may be stronger in those reporting insomnia symptoms compared to those without sleep problems. Notably, previous research on PC and executive functions has focused more on the content of thoughts than on the underlying cognitive processes. For example, in their study, Whitmer and Banich (2007) found that depressive rumination was associated with a deficit in the inhibition of previously relevant information, whereas angry rumination was associated with impairments in the ability to switching to a new task. In last years, however, it became clear that PC is a transdiagnostic factor associated with many psychological disorders (Wahl et al., 2019); namely, the cognitive processes underlying PC are essentially the same across disorder-specific content of cognitions (Ehring et al., 2011). Therefore, more recent research is moving toward the study of the process of PC rather than the content, using content-independent measures of PC. However, research relating the process of PC to executive functions and examining the role of insomnia symptoms is still lacking. Therefore, the aim of this study was to test the effects of PC, as assessed using a content-independent measure and insomnia symptoms on the task-switching paradigm, a widely used experimental task assessing inhibitory and noninhibitory switching capacities (Ballesio et al., 2018a) in a sample of university students.

## Materials and Methods

### Participants

A total sample of 50 participants were enrolled in the study. Participants were unselected university students recruited from the university community of the Faculty of Medicine and Psychology of Sapienza University of Rome through flyers, word of mouth, and announcement during lectures. Participants received no compensation to participate in the study and completed an informed consent prior to the beginning of the study.

### Measures

#### Perseverative Thinking Questionnaire

The Perseverative Thinking Questionnaire (PTQ; Ehring et al., 2011) was used to assess trait PC. The PTQ is a 15-item content-independent measure of PC. It captures cognitive processes underlying PC, including core characteristics of PC (repetitiveness, intrusiveness, and difficulties with disengagement from the thought), perceived unproductiveness of the thought, and mental capacity engaged during PC. Items of the PTQ include “The same thoughts keep going through my mind again and again,” “Thoughts intrude into my mind,” “My thoughts take up all my attention.” In our sample, the PTQ had high reliability (α = 0.95).

#### Insomnia Severity Index

The Insomnia Severity Index (ISI; Bastien et al., 2001) was used to assess the presence and severity of insomnia symptoms (for instance, difficulties falling asleep and/or maintaining sleep, nonrestorative sleep, impact of insomnia on daytime functioning). Insomnia Severity Index score ranges from 0 (insomnia absent) to 28 (very severe insomnia), with 7 as the cutoff for subclinical insomnia and 14 the cutoff for identifying individuals with clinical insomnia in clinical samples. In community samples, a cutoff of 10 is usually suggested to detect individuals with high versus low insomnia symptoms, due to the high sensitivity (86.1%) and specificity (87.7%) of this cutoff in detecting insomnia cases (Morin et al., 2011). In our sample, the ISI had good reliability (α = 0.76).

#### Brief State Rumination Inventory

The Brief State Rumination Questionnaire (BSRI; Marchetti et al., 2019) is a short (eight items) self-reported scale assessing state rumination. In absence of a content-independent measure of state PC, the BSRI was used to control for the effects of state PC on task-switching performance. This was done in line with experimental studies showing that the induction of rumination determines an impairment in task-switching processes (Whitmer and Gotlib, 2012). The BSRI was administered right before task-switching implementation. Items of the BSRI include “Right now, I dwell on negative aspects of myself that I wish I’d stop thinking about,” “Right now, it is hard for me to shut off negative thoughts about myself,” “Right now, I dwell on negative aspects of myself that I wish I’d stop thinking about.” In our sample, the BSRI had high reliability (α = 0.90).

#### Task-Switching Paradigm

**FIGURE 1:**
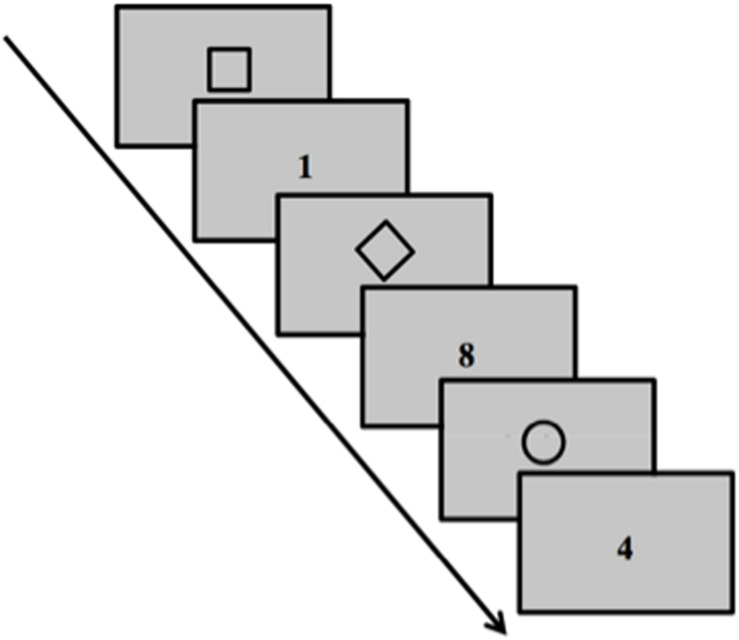
Task Switching paradigm. Schematic representation of task stimuli. The task was composed of 162 trials presented on a 19^″^ computer monitor placed frontally at a distance of about 70 cm.

The task-switching paradigm (Figure 1) was used to assess backward inhibition and switch cost. We followed the same procedure described in detail in Ballesio et al. (2018a, b, 2020). The task is composed of three different rules that need to be applied in random sequences. The concept of backward inhibition involves that switching back to a recently executed task is harder than switching back to a less recently executed task due to residual inhibition. The backward inhibition index reflects slower reaction times (RTs) on the third trial of alternating triplets (A-B-A) versus the third trial of nonalternating triplets sequences (C-B-A) that is due to residual inhibition suffered by rule A in A-B-A versus C-B-A sequences. Moreover, switching from one rule to another implies performance costs, that is, the switch cost. This is reflected in the increase in RTs in switching trials (A-B-A, C-B-A) compared to repetition trials (A-A-A).

### Procedure

Recruited participants completed an informed consent sheet and self-reported questionnaires during an individual laboratory session. Questionnaires included PTQ and ISI and other measures of sleep and cognitive aspects of insomnia disorder not reported in the present study. Following in-laboratory assessment with psychological questionnaires, participants completed the task-switching paradigm assessing executive functions. Right before starting the task, participants completed the BSRI in order to control the presence of state PC. The procedure was approved by institutional review board of the Department of Psychology, Sapienza University of Rome.

### Data Preparation and Analysis

Mean RTs for each sequence (repetition, alternating, switching) of the task switching were collected and computed using SuperLab (Cedrus Corporation, Inc.). Trial errors were not included in the calculations of backward inhibition and switch cost indices. In addition, RTs of ±2 standard deviations from the mean were not included in the calculations. Statistical analysis was computed using IBM SPSS 24. Data were checked for normal distribution (substantial departure from normality was considered as skew value >2 and kurtosis value >7). Descriptive analysis of the sample was performed. One-way analysis of variance (ANOVA) was performed to assess the differences in RTs associated with AAA, ABA, and CBA sequences of the task switching. Furthermore, mixed-design factorial ANOVAs were implemented to examine the interactions between the within-factor task-switching RTs and the between-factor of the group (insomnia vs. good sleepers). Bivariate correlations between the main study variables (PTQ, BSRI, ISI, backward inhibition, switch cost, accuracy) were performed. Furthermore, to estimate the effects of PTQ on task-switching paradigm at different insomnia levels, the sample was split according to ISI scores (Morin et al., 2011) in high insomnia symptoms (ISI > 10) or low insomnia symptoms (ISI < 10), and partial correlations were computed in the two subsamples between PTQ and task-switching indices controlling for the effects of BSRI. Finally, conditional process modeling (Hayes, 2013) was performed to assess the hypothesis that insomnia severity may moderate the relationship between PC and executive functions. In these analyses, PTQ was inserted as independent variable, ISI was inserted as moderator, and task-switching indices were inserted as outcomes. Moreover, to control for the effects of state rumination on task switching, BSRI was inserted as covariate. Two separate analyses were computed for each task-switching outcome (backward inhibition, switch cost). According to our main hypothesis, we expected to find significant PTQ × ISI interactions on backward inhibition and switch cost. Moderation analysis was computed on PROCESS macro for SPSS (Hayes, 2013).

## Results

### Sample Characteristics

Participants were on average 21.85 ± 5.62 years old. Females included in the study were 66%. Participants were all Caucasian. Participants answered correctly at 50.78 ± 5.11 trial on 54 (94.03%). Mean RTs were 715.55 ± 216.09 in the repetition sequences (AAA), 843.02 ± 302.65 in alternating sequences (ABA), and 878.24 ± 292.21 in switching sequences (CBA). Backward inhibition score was on average −30.36 ± 154.87, and switch costs score was 145.08 ± 134.23. Moreover, participants scored on average 7.65 ± 4.27 on ISI, 24.72 ± 13.60 on PTQ, and 215.49 ± 164.46 on BSRI.

### Manipulation Check

One-way ANOVA was performed to assess the differences in RTs associated with AAA, ABA, and CBA sequences of the task switching. Pairwise comparisons showed that AAA sequences were significantly slower with respect to both ABA and CBA sequences (both *p* < 0.01). Reaction times in ABA and CBA were not significantly different (*p* = 0.147). Mixed-design factorial ANOVA examining the interactions between task-switching sequences (RTs in AAA vs. ABA vs. CBA) × group (insomnia vs. good sleep) showed no significant interactions between the two variables (*F* = 1.980, df = 2, *p* = 0.150).

### Correlations

Results on entire sample showed that PC (PTQ) was significantly associated with insomnia severity (ISI, *r* = 0.41, *p* < 0.01) and state rumination (BSRI, *r* = 0.39, *p* < 0.01). Small and marginally significant associations were found between PTQ and backward inhibition (*r* = −0.18, *p* = 0.10) and switch cost (*r* = 0.21, *p* = 0.07). PTQ did not correlate with task-switching accuracy (*r* = −0.08, *p* = 0.28).

To estimate the effects of PTQ on task-switching paradigm at different insomnia levels, the sample was split according to ISI scores in high insomnia symptoms (*n* = 18) or low insomnia symptoms (ISI = 32), and partial correlations were computed in the two subsamples between PTQ and task-switching indices controlling for the effects of BSRI. Results in the group of high insomnia showed that PTQ was significantly and highly correlated with backward inhibition (*r* = −0.57, *p* = 0.01) and with switch cost (*r* = 0.44, *p* = 0.050) but not with task-switching accuracy (*r* = −0.16, *p* = 0.28). Contrarily, in the group of low insomnia, PTQ was not correlated with task-switching performance (backward inhibition: *r* = 0.17, *p* = 0.17; switch cost: *r* = 0.11, *p* = 0.28; accuracy: *r* = 0.07, *p* = 0.36).

### Moderation Analysis

**FIGURE 2:**
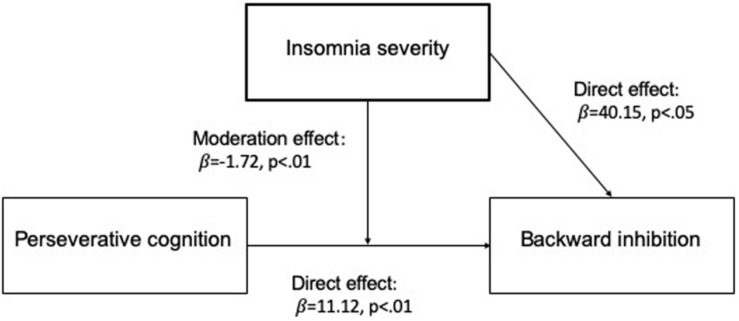
Graphical representation of the moderation model.

A first moderation analysis was performed with PTQ as independent variable, ISI as moderator, and backward inhibition as outcome. In the analysis, BSRI scores were inserted as covariate to control for the effects of state PC on task-switching performance. Results (Figure 2) show that the model was significant (*F*_(4,39)_ = 2.61, *p* < 0.05). Backward inhibition was significantly predicted by PTQ (β = 11.12, *p* < 0.05) and ISI (β = 40.15, *p* < 0.05). Moreover, backward inhibition was predicted by the interaction PTQ × ISI (β = −1.72, *p* < 0.01), with a significantly increased *R*^2^ due to interaction (*R* change = 0.18, *F*_(1,39)_ = 8.72, *p* < 0.05). The effect of BSRI on backward inhibition was not significant (β = 0.06, *p* > 0.05).

A second model was computed with PTQ as independent variable, ISI as moderator, BSRI as covariate, and switch cost as outcome. Results show that the model was not significant (*F*_(4,39)_ = 0.87, *p* > 0.05). Specifically, neither PTQ (β = −1.88, *p* > 0.05), nor ISI (β = −12.71, *p* > 0.05), nor BSRI (β = 0.05, *p* > 0.05), nor PTQ × ISI interaction (β = −53, *p* > 0.05) significantly predicted switch cost. Because the correlation between PTQ and task-switching accuracy was below 0.10 (Cohen, 1992), we did not compute a moderation analysis with accuracy as outcome.

## Discussion

The aim of this study was to test the hypothesis that insomnia symptoms may moderate the relationship between PC and executive functions as assessed using a task-switching paradigm. This paradigm was chosen as a valid tool to study switch cost and backward inhibition in healthy and clinical populations, as documented by several papers from our group (Ballesio et al., 2018a, b, 2020; Cerolini et al., 2020) and other groups (e.g., Sdoia and Ferlazzo, 2008; Couyoumdjian et al., 2010). However, in the present study, we found that while RTs were significantly slower in alternating (ABA) and switching (CBA) when compared to repetition (AAA) trials, reflecting the switch cost, the difference between RTs in ABA and CBA trials was not significant. We explored whether this lack of backward inhibition effect could be due to the clinical condition of the included sample, but mixed-models analysis showed absence of a task × group interaction on RTs. This result is consistent with the findings reported by Philipp and Koch (2006), in which size of *N* − 2 repetition cost (backward inhibition effect) is affected by the occurrence of task repetitions. Taken together, the inconsistencies in the results yielded from studies using this paradigm may reflect the influence on several variables influencing the presence and magnitude of the backward inhibition. However, it was beyond the aim of our study to assess the appropriateness of the task-switching paradigm to detect backward inhibition. Results of the correlations and the moderation models partially support the view that task-switching performance may be influenced by repetitive thought and insomnia. We found that PC is significantly associated with backward inhibition and switch cost in individuals reporting symptoms of insomnia but not in individuals reporting good sleep. Moreover, we found that insomnia moderates the relationship between PC and the performance in the backward inhibition, but not in the switch cost. Findings on backward inhibition are consistent and expand previous literature showing detrimental effects of PC (e.g., rumination, Hilt et al., 2014) and insomnia (e.g., Ballesio et al., 2019) on inhibitory capacities. Moreover, for the first time, here we show that PC and insomnia interact in influencing inhibitory control processes involved in the task switching. Specifically, results show that the negative effects of PC on inhibitory capacities increase at increasing levels of insomnia symptoms.

Results also show that neither PC (neither assessed with a trait nor with a state measure) nor insomnia was associated with switch cost in moderation analysis. This result is in contrast with a recent meta-analysis finding a positive correlation between PC (rumination) and switching capacities (Yang et al., 2016). However, this is consistent with a previous experimental study using the task-switching paradigm and showing that inducing rumination impacts switch cost in individuals with major depression but not in healthy controls (Whitmer and Gotlib, 2012). This is also consistent with a previous study showing that depressive rumination is associated with backward inhibition but not with noninhibitory switching abilities (Whitmer and Banich, 2007). Also, it must be noted that, in contrast with previous literature, we studied PC using a content-independent measure. Specifically, the cognitive correlates of the PTQ have never been investigated to date. It is therefore possible that this specific instrument may have limited sensitivity in detecting noninhibitory switching abilities.

### Clinical Implications

Results from the present study may suggest several clinical implications. Both PC and insomnia are considered as transdiagnostic factors associated with several psychopatho- logical conditions including mood and anxiety disorders, obsessive-compulsive disorders, posttraumatic stress disorders, and personality disorders and psychosis (Harvey, 2008; Wahl et al., 2019). Importantly, meta-analyses show that these disorders are associated with moderate–large inhibitory control impairments (Snyder et al., 2015). Therefore, if confirmed with longitudinal or experimental studies in clinical populations, our findings would highlight the importance to target both PC and insomnia in clinical interventions in order to improve cognitive functioning. Preliminary evidence, for instance, suggests that behavioral treatment of insomnia has a moderate impact on PC (e.g., Ballesio et al., 2018b; van der Zeerde et al., 2019). Similarly, clinical protocols for reducing form of PC, such as cognitive behavioral therapy for worry, are associated with a reduction in insomnia symptoms (Bush et al., 2012). However, randomized controlled trials exploring the mediation effects of insomnia and PC on cognitive functioning are lacking and therefore encouraged.

### Limitations

Several limitations need to be acknowledged. As the first study exploring the combined effect of PC and insomnia on task switching, we adopted a cross-sectional design. However, to infer stronger causal relationships between variables under study, future research is strongly recommended to employ experimental and longitudinal designs. Furthermore, we assessed *n* − 1 switch and *n* − 2 repetition cost in a single task-switching session, which may reduce the involvement of inhibitory processes (Hartmann et al., 2019). Moreover, our study comprised a convenience and small sample of university students. Notably, university students are particularly at risk of suffering from insomnia due to stress and academic demands and dysfunctional sleep habits (e.g., late-night computer work, substance use, irregular bed timing) (Jiang et al., 2015). Specifically, a recent meta-analysis of epidemiological literature comprising 16,478 participants estimated the prevalence of insomnia at 18.5% of university students. However, our sample was unselected, and therefore future larger studies are needed to replicate our findings in clinical samples in order to increase the generalizability of our findings.

## Data Availability Statement

The datasets generated for this study are available on request to the corresponding author.

## Ethics Statement

The studies involving human participants were reviewed and approved by Institutional Review Board Department of Psychology, Sapienza University of Rome. The patients/participants provided their written informed consent to participate in this study.

## Author Contributions

AB planned the experiment, ran the analysis, and wrote the manuscript. MV and SC helped with the data analysis. FL and CL revised the final draft of the manuscript. All authors contributed to the article and approved the submitted version.

## Conflict of Interest

The authors declare that the research was conducted in the absence of any commercial or financial relationships that could be construed as a potential conflict of interest.
